# Stick, Slide, or Bounce: Charge Density Controls Nanoparticle Diffusion

**DOI:** 10.1021/acsnano.4c05077

**Published:** 2024-10-08

**Authors:** Ahmad Reza Motezakker, Luiz G. Greca, Enrico Boschi, Gilberto Siqueira, Fredrik Lundell, Tomas Rosén, Gustav Nyström, L. Daniel Söderberg

**Affiliations:** † Department of Engineering Mechanics, KTH Royal Institute of Technology, Stockholm, SE 100 44, Sweden; ‡ Wallenberg Wood Science Center, KTH Royal Institute of Technology, Stockholm, SE 100 44, Sweden; § Laboratory for Cellulose and Wood Materials, Swiss Federal Laboratories for Materials Science and Technology (Empa), Dübendorf 8600, Switzerland; ∥ Department of Fibre and Polymer Technology, KTH Royal Institute of Technology, Stockholm, SE 100 44, Sweden; ⊥ Department of Health Sciences and Technology, ETH Zürich, Zürich 8092, Switzerland

**Keywords:** nanoparticle diffusion, electrostatic interactions, polymer networks, controlled release, surface charge effects, molecular dynamics simulations, drug delivery

## Abstract

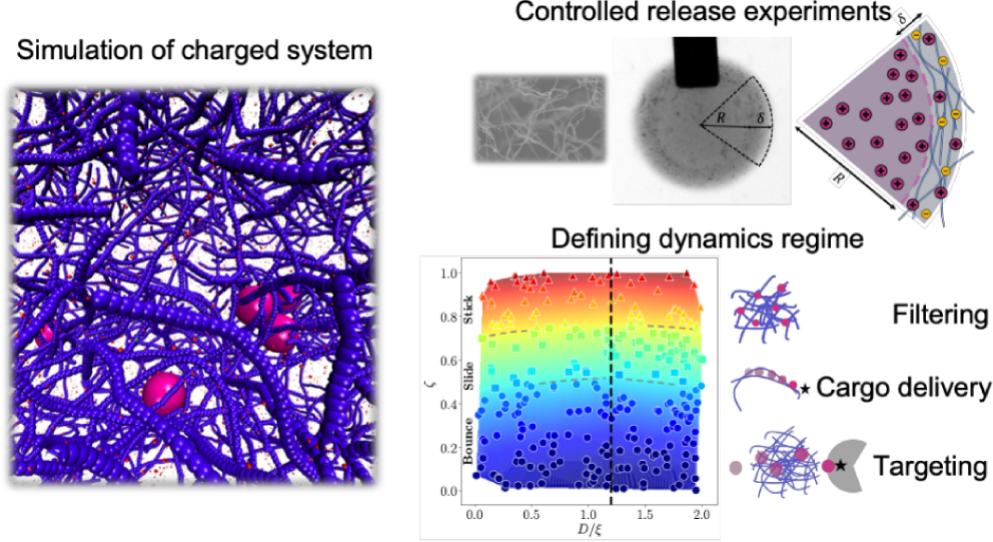

The diffusion and interaction dynamics of charged nanoparticles (NPs) within charged polymer networks are crucial for understanding various biological and biomedical applications. Using a combination of coarse-grained molecular dynamics simulations and experimental diffusion studies, we investigate the effects of the NP size, relative surface charge density (ζ), and concentration on the NP permeation length and time. We propose a scaling law for the relative diffusion of NPs with respect to concentration and ζ, highlighting how these factors influence the NP movement within the network. The analyses reveal that concentration and ζ significantly affect NP permeation length and time, with ζ being critical, as critical as concentration. This finding is corroborated by controlled release experiments. Further, we categorize NP dynamics into sticking, sliding, and bouncing regimes, demonstrating how variations in ζ, concentration, and NP size control these behaviors. Through normalized attachment time (NAT) analyses, we elucidate the roles of electrostatic interactions, steric hindrance, and hydrodynamic forces in governing NP dynamics. These insights provide guidance for optimizing NP design in targeted drug delivery and advanced material applications, enhancing our understanding of NP behavior in complex environments.

## Introduction

Engineered nanoparticles (NPs) can serve as a link between materials science and biophysics, significantly advancing the functionality and efficiency of diagnostic and targeted drug delivery systems.^1^ They are able to move through various systems—like webs or matrices—to deliver, activate, or accumulate targeted substances. The process of nanoparticle diffusion in networks, such as biological hydrogels, is complex and is influenced by several interacting factors. Key mechanisms encompass steric hindrance, influenced by the nanoparticle’s size, and the density of the surrounding medium, along with hydrodynamic effects and electrostatic interactions. Additionally, the adaptability and dynamic reconfiguration of the surrounding medium further amplify this complexity.^2,3^ The impact of each factor varies depending on the specific area of research and application. Notably, electrostatic interactions play a crucial role in biological contexts, driving the development of sophisticated engineered particles for diverse applications, including diagnostics and cancer therapy,^4−6^ immune system modulation,^7,8^ and gene therapy.^9−11^

Despite advancements in charge tunability for NPs, challenges persist across various microenvironments. Both positively and negatively charged particles show reduced diffusivity in extracellular matrix (ECM) gels, regardless of the ECM’s net negative charge. This underscores complex biological interactions and the barriers to effective diffusion.^12^ Viruses, even when smaller than mucus pores, can become trapped, highlighting the intricate nature of biological barriers.^13,14^ The local microenvironment significantly affects NPs’ therapeutic and diagnostic efficiency. For example, tumor microenvironments, characterized by excessive ECM production and abnormalities in lymphatic vasculature, restrict NP delivery and perfusion, particularly for cationic NPs that bind to the negatively charged tumor ECM.^15−20^ Furthermore, the mucus layer’s variable mesh pore sizes and propensity for nonspecific interactions complicate NP diffusion and can result in rapid clearance from epithelial surfaces.^21^ This complexity contributes to the fact that, on average, only 0.7% of administered NPs successfully reach tumor sites, underlining a critical gap in our understanding and their application in biomedical contexts.^19^ Addressing these challenges through detailed research on NP transport and interaction mechanisms, especially in charged environments, could significantly advance nanoparticle-based therapies, marking a leap forward in biomedical technology and patient care.

Recent studies have made significant progress in understanding how the dynamic properties, flexibility, and topology of polymer networks,^22−24^ as well as the shape and rigidity of NPs themselves,^25,26^ influence NP diffusion. Building upon these results, this study combines large-scale coarse-grained simulations and experimental methods to investigate how NPs move and distribute across different environments, focusing on the influence of particle size, hydrodynamic forces, and the specific effect of electrostatic interactions. Leveraging on insights from previous studies,^27^ we model the diffusion of charged and neutral NPs within charged polymer networks to encompass a variety of charge combinations and network conditions. These models include several layers of complexity including the emergent structure of the fibrous network from NP, fiber, and counterion interactions under varying degrees of crowding. Experimentally, we assess the spread of charged NPs through a negatively charged cellulose nanofiber (CNF) network, which also serves as a model for biological hydrogels in drug delivery,^28,29^ by testing different NP sizes and dye types at several concentrations. We elucidate diffusion behaviors and patterns to correlate these experimental results with our simulation insights, enhancing our understanding of the NP dynamics in complex matrices. This comprehensive analysis helps bridge the gap between theoretical predictions and real-world observations, contributing to developing superior drug delivery as well as analytical and material science systems. Apart from diffusivity, the analysis can show under which conditions the NPs experience one of the following dynamic events: sticking, sliding, and bouncing.

## Results and Discussions

**Figure 1:**
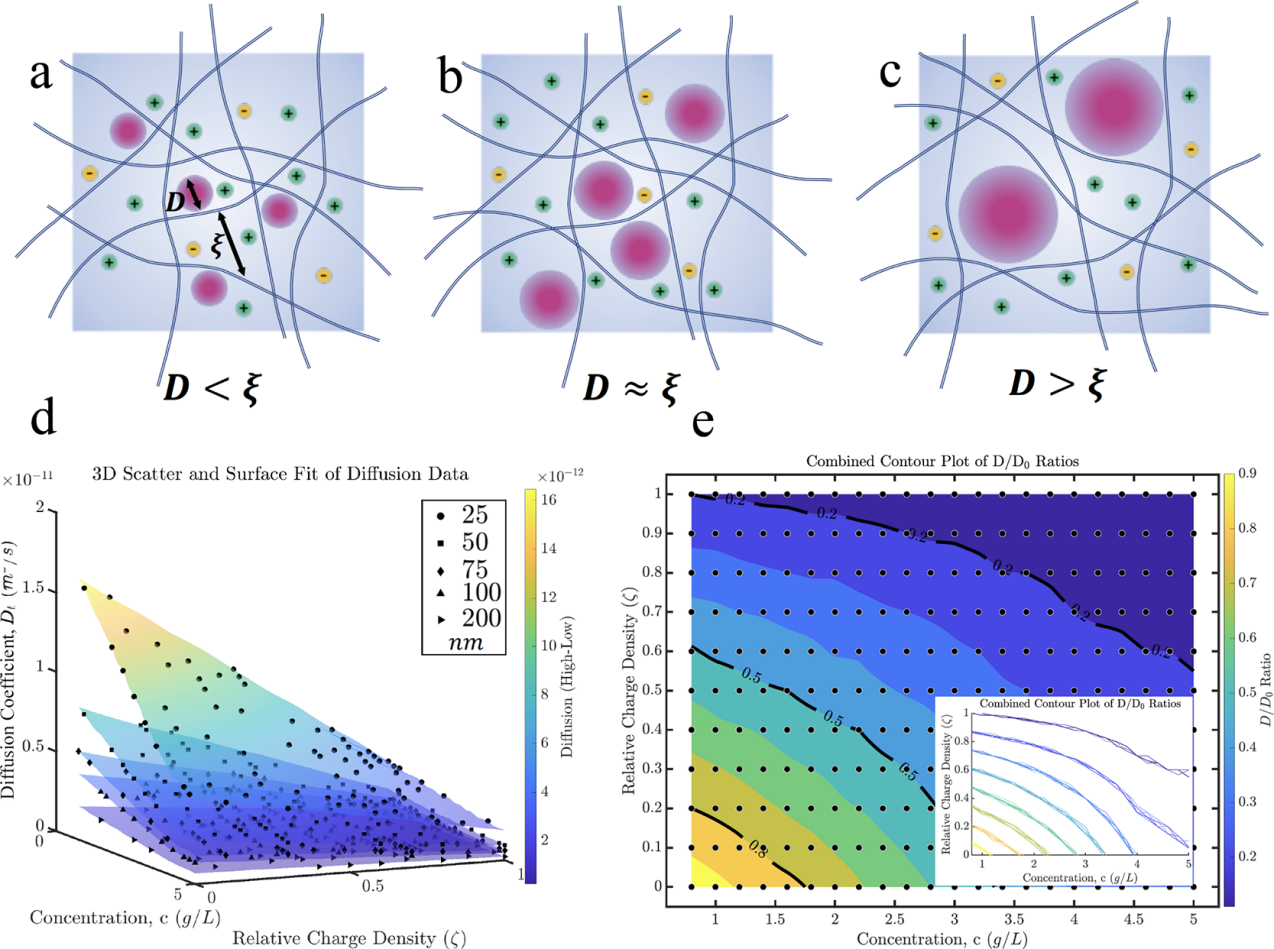
Schematic and analysis of particle diffusion in a polymer network. Panels (a–c) depict schematics of particles of size *D* within a polymer network characterized by a mesh size of ξ, illustrating scenarios where *D*<ξ (a), *D* ≈ ξ (b), and *D*>ξ (c), respectively. The bluish color of the fibers indicates they are negatively charged, while the pinkish color of the nanoparticles indicates they are positively charged. The other charges shown are counterions. Panel (d) presents a 3D scatter plot alongside a surface fit of diffusion data, highlighting how nanoparticle (NP) diffusion diminishes with increasing concentration and relative surface charge density, with a pronounced decrease observed for smaller particles. Panel (e) features a contour plot of the normalized diffusion coefficient as a function of concentration and relative charge density for the case of an NP size of 25 nm, with an inset showing isolines for various NP sizes, indicating a convergence of diffusion characteristics across different sizes.

To study the dynamics of NPs through complex environments, we rely on a combination of simulations and experimental studies (for details on the simulations, see Supporting Information). We primarily investigate the impact of three key parameters: the NP diameter *D*, the mesh size of the network ξ, and the relative surface charge density ζ. Specifically, ζ is the absolute value of the ratio of the surface charge density of NPs to that of oppositely charged nanofibers. Without loss of generality, we here discuss the case of positively charged NPs and negatively charged nanofibers. We have studied NP diameters ranging from 25 to 200 nm, network concentrations between 0.8 and 5 g/L (0.08 and 0.5 wt %), and relative surface charge densities from zero to 1. This range of parameters was chosen to encompass various scenarios that might influence the behavior of NPs relative to the surrounding fibrous concentration and their relative surface charge. Figure 1a–c depicts how the size of the particles, whether smaller, similar-sized, or larger than the mesh size, alongside the charge of the system, determines the particles’ behavior, from free diffusion to altered movement and also sticking to nanofibers due to electrostatic attraction.

Figure 1d shows a three-dimensional plot of NPs’ diffusion coefficient (*D*_t_) against the concentration of the fibrous network and relative surface charge density for various NP sizes. For all the cases *D*_t_ has been calculated with Green–Kubo relation, , where *v*(*t*) is the velocity of a particle at time *t*, and ⟨·⟩ denote the ensemble average over all particles and initial times. This graph demonstrates the individual and combined effects of the concentration and charge on the diffusion process, as indicated by the surfaces fitted to the data points. Notably, with increasing concentration and relative charge, *D*_t_ diminishes. This trend is more pronounced for smaller NPs than for larger ones. This can result from lower mass, making smaller NPs highly sensitive to hydrodynamic interactions, especially in denser fibrous networks or those with higher relative charge. Although it is commonly accepted that particles smaller than the mesh size, *D* < ξ, can freely diffuse within the system, the data suggests that the decrease in diffusion starts before reaching the point where *D* ≈ ξ, attributed to the electrostatic attraction between the nanoparticles and the fibers. Depending on the charge disparity, NPs might either permanently stick to or slide along the fibers. In both scenarios, the movement of the nanoparticles is significantly restricted, leading to the observed decrease in *D*_t_.

Interestingly, when *D*_t_ is normalized by *D*_0_, the diffusion coefficient of NPs in a dilute environment, and plotted as contour levels versus the relative charge of the system and concentration (Figure 1e), all cases collapse to the same isovalue lines. Normalizing the diffusion coefficients of nanoparticles in a fibrous network by their diffusion in water, *D*_0_, accounts for inherent differences in mobility due to particle size, allowing a size-independent analysis of how fiber concentration and electrostatic interactions impact diffusion. This normalization reveals a universal scaling behavior: despite smaller particles experiencing a stronger absolute reduction in diffusion due to forces, the relative impact of the network and interactions—when measured against each particle’s baseline mobility in water—demonstrates a consistent pattern across all sizes. This implies that proportionally, the presence of fibers and changes in ζ affect nanoparticle diffusion similarly, regardless of their size. The normalization thus clarifies the initial discrepancy between absolute and relative changes in diffusion, revealing that the intricate dynamics governing nanoparticle movement in fibrous environments adhere to universal principles that emerge when examining both absolute and relative diffusion measures.

When it comes to the diffusion of NPs, there are indeed several well-established theories available.^30−32^ However, like many other models, these theories are based on specific assumptions, such as treating the polymer network as a static, motionless structure with repetitive, defined patterns, typically excluding electrostatic interactions. One of the recent studies introduces a multiscale model for NP diffusion in polymer networks, combining three key frameworks—hydrodynamic, free volume, and obstruction theory—into a comprehensive approach known as the multiscale diffusion model (MSDM).^33^ The fitted lines from the simulation results are compared with the MSDM, free volume, and obstruction theories for mesh sizes of ξ = 30, 20, and 10 nm across various charge ratios (see Figure S6). In scenarios with no electrostatic interactions (ζ = 0), the simulation results align well with the MSDM theory for *D*/ξ > 1. However, for *D*/ξ < 1, deviations occur, likely due to the influence of network dynamics on NP behavior, as the theories assume a static network with well-defined structures. For cases with ζ = 0.5 and 1, the results show greater deviation from the MSDM theory, eventually becoming almost compatible with the MSDM theory for *D*/ξ > 2. In this regime, the NP size becomes significantly larger than the mesh size, making the mesh size the limiting factor and thereby dominating electrostatic interactions.

The inherent complexity of polymer networks and biological systems challenges the development of theoretical frameworks for diffusion. Despite this, several theories have emerged to understand and predict diffusion behavior, leading to semi-empirical scaling laws that account for key parameters.^34−36^ These laws often exhibit a stretched exponential form, expressed as , where *c* is the polymer network concentration, α is a scaling factor, and *v* characterizes the concentration dependence. In the literature, *v* values range from 0.5 to 1 under various conditions.^34−38^ To incorporate the influence of ζ, the expression can be modified, acknowledging the interdependent effects of the concentration and charge on diffusion. The revised scaling law is expressed as . This formulation maintains the stretched exponential behavior and allows us to account for the significant impact of the relative charge on diffusion besides concentration. By fitting the data to this modified equation, we reach *v* = 0.602 ± 0.012 and β = 0.518 ± 0.015.

**Figure 2:**
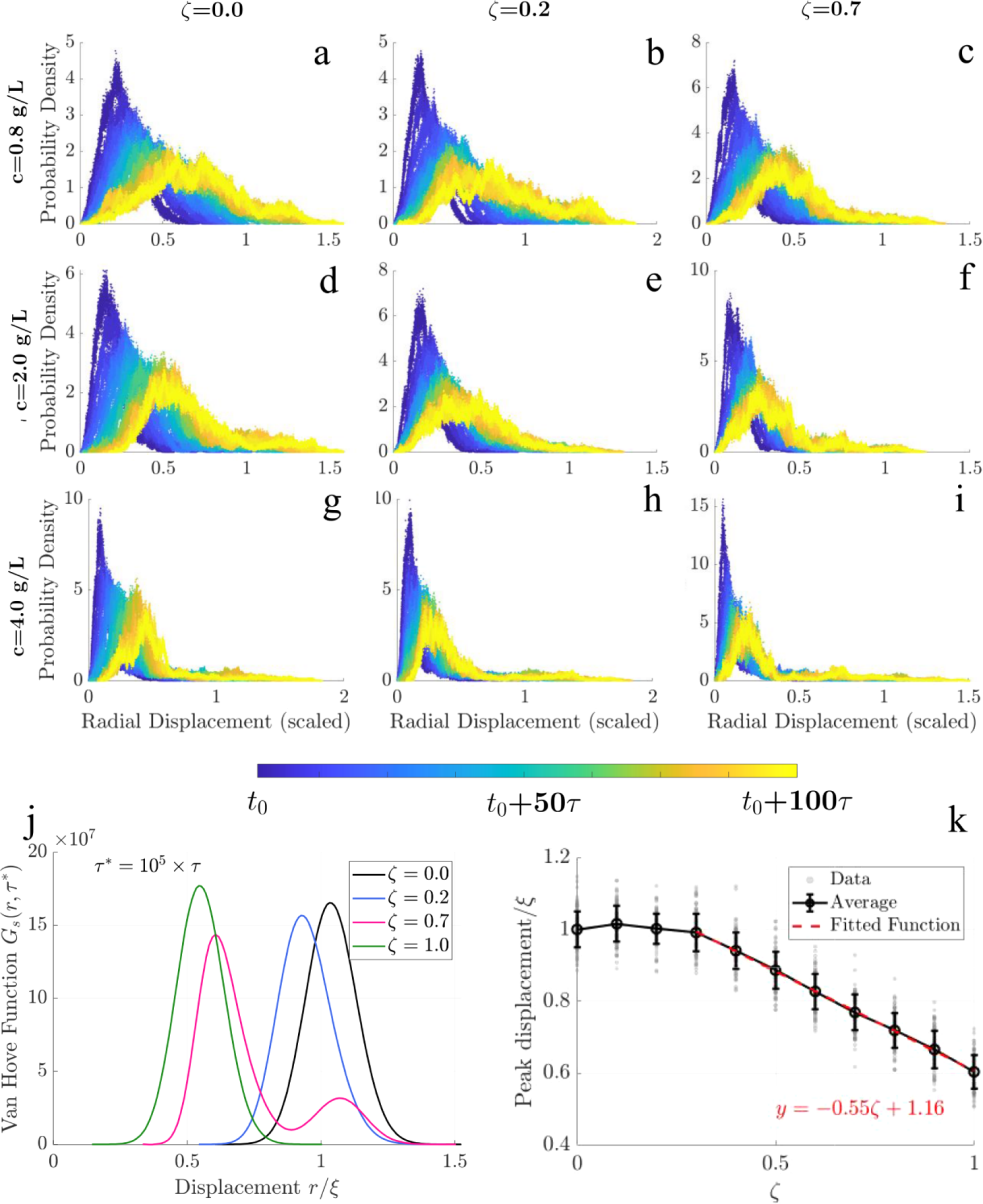
(a–i) Probability density functions (PDFs) of nanoparticles (NPs) within a network, categorized by relative charge densities (ζ) and concentration levels. Rows represent increasing concentrations, while columns correspond to ζ values of 0 (neutral NPs), 0.2, and 0.7, illustrating the distribution of radial displacements across different charge states and concentrations. (j) Van Hove analysis for a low concentration case (*c* = 0.8 g/L) with ζ values of 0.0, 0.2, 0.7, and 1. For ζ = 0.7, the peak shifts significantly to the left, corresponding to a displacement of *r*/ξ = 0.6. Additionally, the Van Hove plot exhibits two peaks, indicating a bimodal distribution. (k) Impact of relative surface charge density on the peak displacement of the NP distribution.

Figure 2a–i illustrates the impact of polymer concentration and charge on the NP distribution. Each row represents a different polymer concentration (0.8 g/L to 4 g/L), and columns represent ζ values (neutral ζ = 0, moderately charged ζ = 0.2, and highly charged ζ = 0.7). At 0.8 g/L, PDFs show a broader NP distribution, indicating more dispersion due to fewer obstacles. As concentration increases, PDFs narrow, signifying a more constrained NP distribution. At 4 g/L, NPs show a pronounced peak, reflecting substantial accumulation.

Comparing neutral (ζ = 0) and charged NPs (ζ = 0.2, 0.7), a clear shift in peak and NP accumulation is observed. Neutral NPs have a broad distribution across all concentrations, with slight accumulation as the concentration increases. Charged NPs display sharper peaks, especially at higher concentrations, indicating stronger electrostatic interactions and more significant accumulation, most pronounced for highly charged NPs (ζ = 0.7). To analyze the effect of the charge, further analysis was conducted to relate the peak displacement in the PDFs to the mesh size of the system. Specifically, for a low concentration case of *c* = 0.8 g/L, Van Hove analysis was performed for three cases: ζ = 0.0, 0.2, and 0.7. As illustrated in Figure 2j, the Van Hove function *G*_s_(*r*, τ*) is plotted at the same concentration and long-term time lag of τ* = 10^5^ × τ, with the displacement *r* normalized to the mesh size of the system to emphasize the relationship. As expected, in the case of ζ = 0, where the NPs are neutral, the peak displacement is approximately *r*/ξ ≈ 1. This indicates that without attraction between the NPs and the polymer chains, the NPs eventually become trapped in the system in a cage with a size corresponding to the system mesh. As ζ increases, the peak shifts to the left. For ζ = 0.2, the peak displacement *r*/ξ becomes smaller than 1. Although the tail of the distribution extends to values larger than 1, it indicates that most NPs cannot permeate distances equal to the mesh size due to the slight attraction between NPs and polymer chains. For ζ = 0.7, the peak shifts significantly to the left, corresponding to a displacement of *r*/ξ ≈ 0.6, and the Van Hove plot results in a bimodal distribution. In these cases, with approximately 90% of the NPs positioned before the secondary peak, the focus remains on the primary peak as this approach effectively isolates the charge-induced displacement changes from the baseline behavior dictated by the network mesh size. In scenarios with moderate ζ, where the sliding regime is dominant, it is hypothesized that NPs move along the polymer chains, with some eventually becoming trapped in the mesh structures, leading to the observed secondary peak. For cases with high ζ values (ζ = 1), the behavior reverts to a single peak.

To further analyze the effect of ζ, we identified the dominant peaks and their corresponding displacements for all cases were identified. Figure 2k, shows the relative peak displacement to the mesh size plotted against ζ. The average peak displacement relative to ξ remains nearly equal to 1 up to ζ ≈ 0.25. For ζ values greater than 0.25, the peak displacement becomes significantly smaller than the mesh size. To quantify this behavior, a line was fitted to the average values of peak displacement/ξ, resulting in
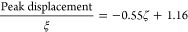


**Figure 3:**
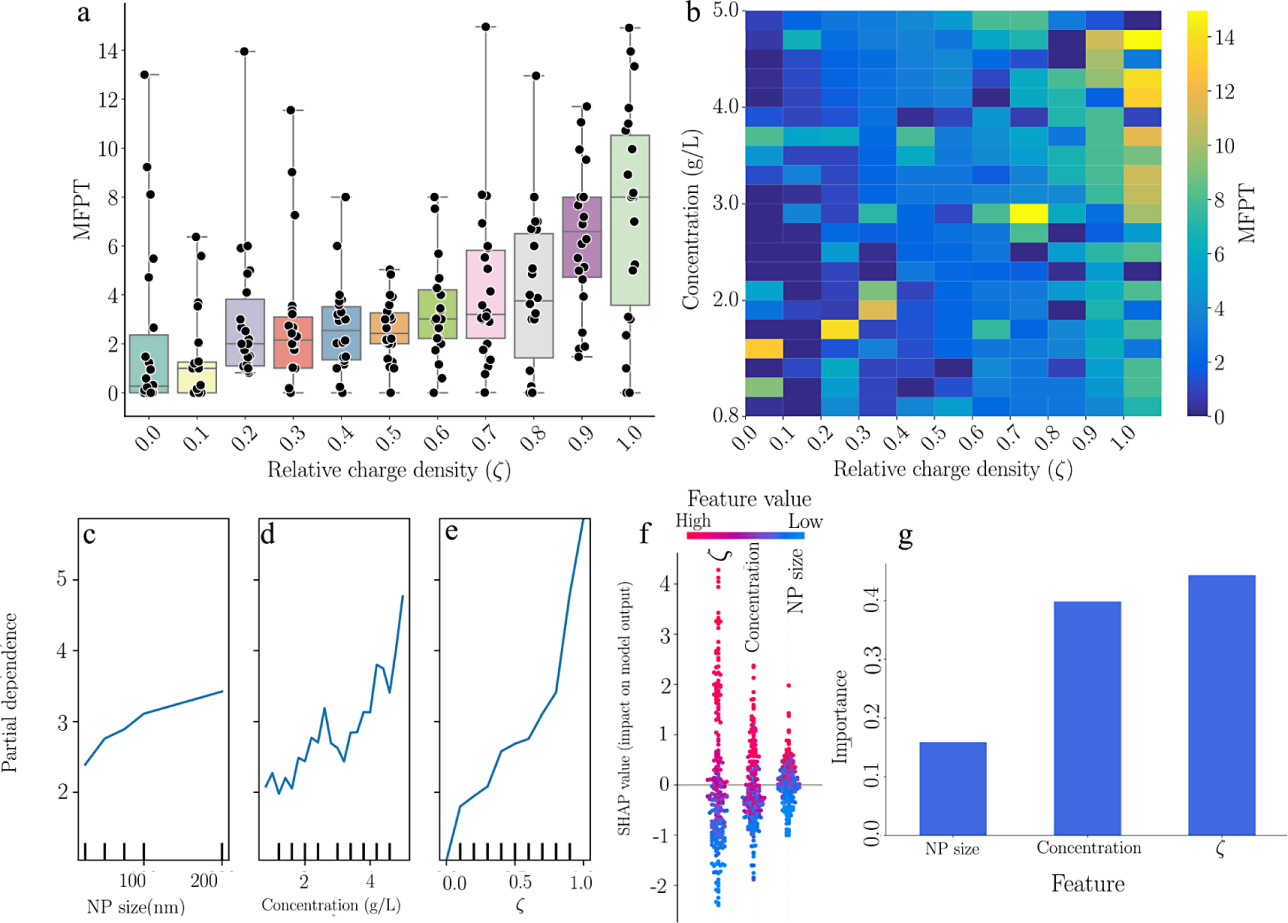
Comprehensive mean first passage time (MFPT) analysis concerning nanoparticle characteristics and polymer network parameters. (a) Box plots showing the distribution of MFPT for different relative charge densities (ζ). (b) Heatmap showing the MFPT for a combination of polymer concentration (g/L) and ζ. (c–e) Partial dependence plots for NP size, concentration, and ζ demonstrate their influence on MFPT. (f) SHAP (Shapley additive explanations) values reveal the distribution of impacts from each feature value on the model’s prediction. (g) Feature importance bars indicate the relative predictive power of NP size, concentration, and ζ within the random forest model.

Following the spatial analysis of NPs through PDFs in Figure 2, the analysis is extended by considering the temporal dynamics, which is pivotal to gaining a thorough understanding of the NP behavior within polymer networks. This analysis is critical because, while PDFs reveal the spatial distribution and localization of NPs, they only partially capture the temporal aspect of NP movement through the polymer network. To address this, the mean first passage time (MFPT) is chosen as a complementary metric, offering insights into the temporal efficiency of NPs in reaching specific regions within the network. In essence, the MFPT quantifies the time it takes for a particle to arrive at a designated target location, serving as a vital measure of the dynamic interactions and mobility within complex systems. In Figure 3a, the box plots of MFPT show a complex trend across different ζ, with MFPT varying across ζ values. A wide range of MFPT values at intermediate ζ indicates diverse particle transit times. Higher ζ values generally correspond to higher MFPT, suggesting that increased electrostatic interactions may slow the NP movement. However, the pattern is inconsistent, indicating that factors beyond charge density, such as the dynamic structural features of the polymer network, significantly influence NP mobility. If the network structure was predefined and repeating,^22^ the outcomes could show a consistent pattern, different from current observations, due to nanoparticles moving directly from one cage to another, with each cage ensuring capture. In Figure 3b, the heatmap shows how MFPT varies with concentration and charge density in the polymer network. The heatmap reveals that MFPT does not uniformly increase with concentration or charge density but instead shows notable variations across specific combinations, represented by contrasting colors. The heatmap highlights the system’s complexity, where dynamic polymer network arrangements and electrostatic charge distributions lead to diverse NP interactions and variable MFPT.

Due to the system’s complexity, we employ random forest models with SHAP (Shapley additive explanations) to classify complex data relationships. Random forests handle large data sets and show interactions without needing a specific model. SHAP values clarify predictions by showing each feature’s contribution. This combination is particularly useful for understanding factor interactions in complex systems. The partial dependence plots (Figure 3c–e) reveal insights into the NP motion. A nearly linear relationship with NP size suggests larger NPs navigate more slowly due to increased obstruction. The relationship between concentration and MFPT shows high variability, indicating varying NP mobility influenced by network dynamics. A sharp increase in MFPT at higher ζ values highlights the significant impact of electrostatic forces on NP movement. To visually demonstrate the significance of electrostatic forces, Movie S1 illustrates the diffusion of an NP within a dynamic mucus structure. Despite being smaller than the mesh size (*D* < ξ), the NP can adhere to the mucus and become trapped, aligning with previous studies.^13,14,39,40^

Figure 3f shows SHAP values for NP size, concentration, and relative charge density using a color gradient from blue to red for low to high feature values. The vertical spread of SHAP values reflects their impact on predicting MFPT, with higher values indicating longer MFPT. Concentration and ζ exhibit broader spreads, suggesting a more complex, nonlinear influence on MFPT, while NP size shows a narrower distribution, indicating a consistent contribution. Larger NP sizes modestly increase MFPT, while higher ζ values significantly increase MFPT due to stronger electrostatic interactions. Figure 3g presents a bar chart quantifying the importance of each feature in the random forest model and shows how each feature contributes to predictive accuracy. The chart reveals that concentration and relative charge density (ζ) are more influential than NP size in predicting MFPT. This indicates that variations in MFPT are more responsive to changes in concentration and ζ than NP size. This result shows that relative surface charge density is as crucial as concentration in determining the spread time of NPs. For instance, in designing a controlled release system with low polymer concentration, our results indicate that adjusting the charge can regulate the release time without altering the polymer concentration. This finding highlights the significance of electrostatic interactions in the design of NP-based drug delivery systems.

To evaluate the significance of the simulations, experiments have been designed to elucidate the mechanisms controlling this complex system. In the experiments, two types of positively charged dyes, thionine and methylene blue, and one type of negatively charged NP dye, Congo red, are systematically released into a negatively charged biopolymer network. TEMPO-oxidized cellulose nanofiber gel networks have been used, leveraging its potential as a model for biological hydrogels^28,29^ and aligning with the negatively charged characteristics of biological networks like the extracellular matrix (ECM).^41,42^

**Figure 4:**
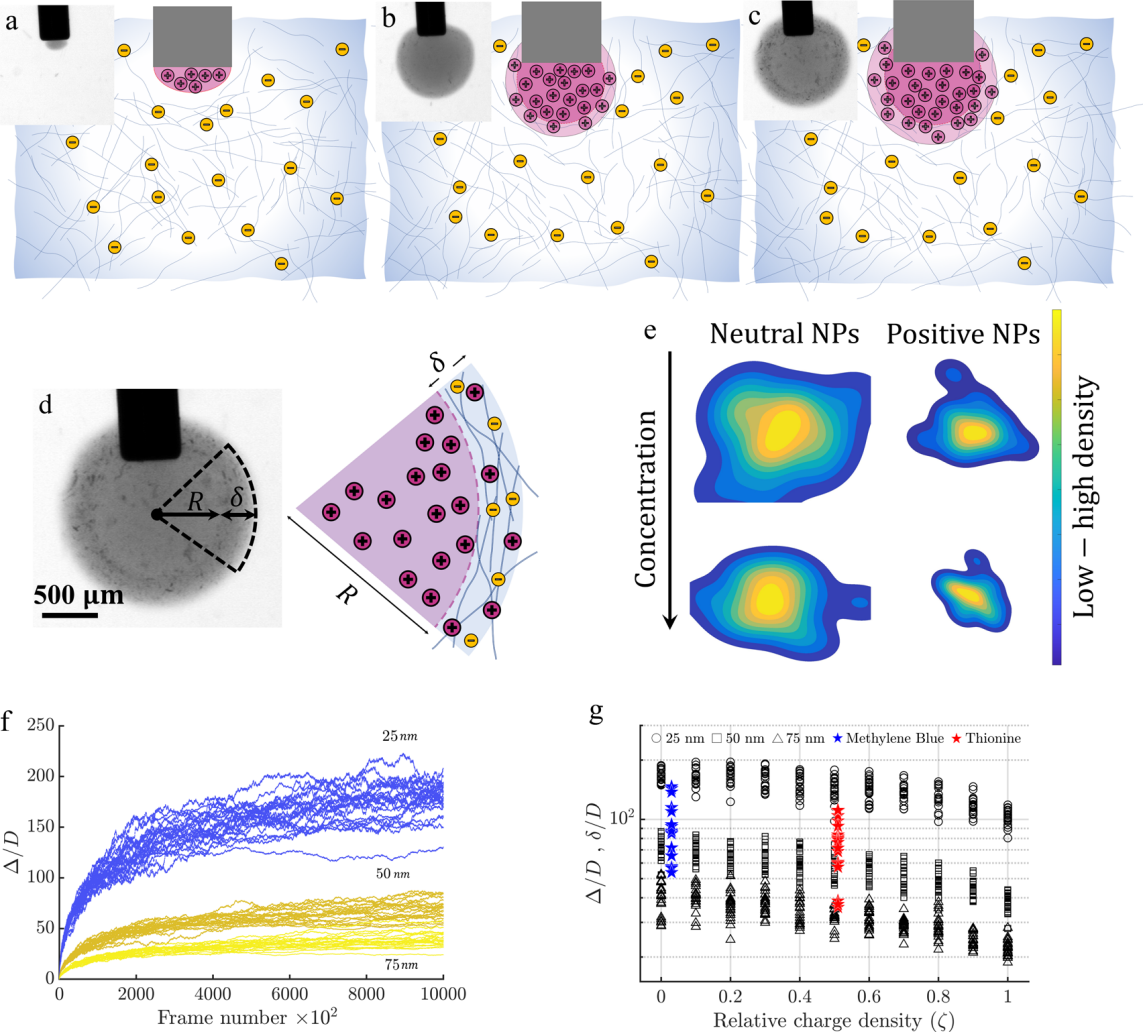
Experimental observations and simulation analysis of dye release in biopolymer suspensions. Panels (a–c) capture experimental snapshots and corresponding schematics illustrating the release of 1 μL of 3 mM thionine into a cellulose nanofiber suspension with a concentration of 0.8 g/L, detailing the time-evolution of complexation. Panel (d) zooms into the complexation structure, showcasing the permeation length of dye nanoparticles alongside a detailed schematic. Panel (e) features a combined kernel density estimate (KDE) plot from simulations, elucidating the impact of nanoparticle concentration and charge on permeation length and distribution symmetry. Panel (f) traces the growth and stabilization of permeation lengths over simulation frames for nanoparticle sizes of 25, 50, and 75 nm. Lastly, panel (g) compares permeation lengths derived from simulations and experiments against relative charge density.

Movies S2 and S3 depict the process of dye release in a network over a 2 min period. In Movie S2, an illustration of releasing 1 μL of 3 mM thionine into a cellulose nanofiber suspension with a concentration of 0.8 g/L, accompanied by its image analysis, is provided. As anticipated for Congo red, which is negatively charged, no notable observations were made. Detailed information regarding the experiments, setup, and postanalysis can be found in the Supporting Information. In Figure 4a–c, experimental images along with their corresponding schematics are presented. Figure 4a illustrates the initial presence of the positive dye, thionine, within the TEMPO-oxidized CNF network. At this stage, a membrane-like structure forms due to the complexation of positive and negative charges, expanding in a circular pattern until it nearly matches the diameter (*R*) corresponding to the volume of dye released (Figure 4b). Understanding and controlling such complexation process is key for the development of cancer therapies relying on the in situ formation of hydrogels for targeted drug delivery.^43^ The stability of the membrane varies depending on the disparity in charge density between the positive dye and the CNF concentration. In instances of low to moderate charge density differences, the circular membrane typically remains stable, while in cases of high charge density differences, the membrane ruptures (see Movie S4). After this stage, positive molecules diffuse into the CNF environment, as evidenced in Figure 4c and detailed in Figure 4d. The distance that molecules can permeate through the system is termed δ, and this distance is dependent on size, charge, and surrounding charge density. The length of δ is the most crucial parameter for this aspect of the experiments.

To gain further insight into δ and to compare with the experiments, multiple simulations were conducted, wherein the origins of all NPs were shifted to a common point for analysis. This approach was chosen over releasing all particles at the network center due to simulation constraints and forces involved, making the latter practically unfeasible. In Movie S5, a detailed observation reveals how the concentration of the polymer network and the positive charge of NPs influence the dispersion and symmetry of NPs within the system. Figure 4e provides a snapshot of the kernel density estimate (KDE) plot for four scenarios (low and high concentrations and neutral and positive charge of NPs). Initially, it is evident that the spread range of NPs is wider for low concentration and neutral NPs, while it is narrower for high concentration and positively charged NPs. Furthermore, the impact of the charge on the symmetry and skewness of the distribution is notable, stemming from the attraction between oppositely charged NPs and the network. Notably, in Movie S1, as time progresses, the influence of opposite charges becomes apparent on the dynamics of NPs, with a noticeable decrease in the rate of change in distribution shape, indicating the entrapment and settling of NPs onto the polymers.

In the simulations, the average distance traveled by all NPs at each frame is referred to as Δ. Figure 4f illustrates the scaled Δ with NP diameter for three NP sizes plotted against frame numbers. It is clear that for all cases, Δ shows an asymptotic trend, where the asymptotic behavior to some extent can be compared to δ observed in experiments. The reasoning behind this is that both represent the permeation length of positive particles within the system before they attach to the network, forming complexations. In Figure 4g, the normalized permeation lengths, Δ and δ, are plotted against relative charge density. For the experimental results involving Thionine and Methylene Blue, aimed at estimating ζ and the size of aggregated dyes, certain assumptions were made to correlate the zeta potential (ζ_P_) and mobility with these parameters (see Supporting Information). It is clear that the permeation lengths decrease with an increase in ζ, a trend anticipated due to the enhanced electrostatic attraction between the nanoparticles (NPs) and the network. The similar downward trajectories for both δ and Δ as a function of ζ are notable. Thus, it can be inferred that the normalized permeation lengths diminish with ζ, adhering to a consistent trend. At this stage, it is crucial to understand the mechanisms occurring within the charged systems that affect the permeation lengths for the NPs in various microenvironments. Various simulation scenarios have therefore been carefully visualized to assess the impacts of NP size and ζ on the mobility of NPs. This involves examining how NPs are attracted to the dynamic polymers, whether they stick to them or slide along them, and how these motions affect the structure of the network. In Movies S6, S7, S8, and S9, four scenarios are presented, encompassing two cases of particle size (*D* < ξ and *D* = ξ) and two cases of relative surface charge density (ζ = 0.2 and ζ = 0.7).

**Figure 5:**
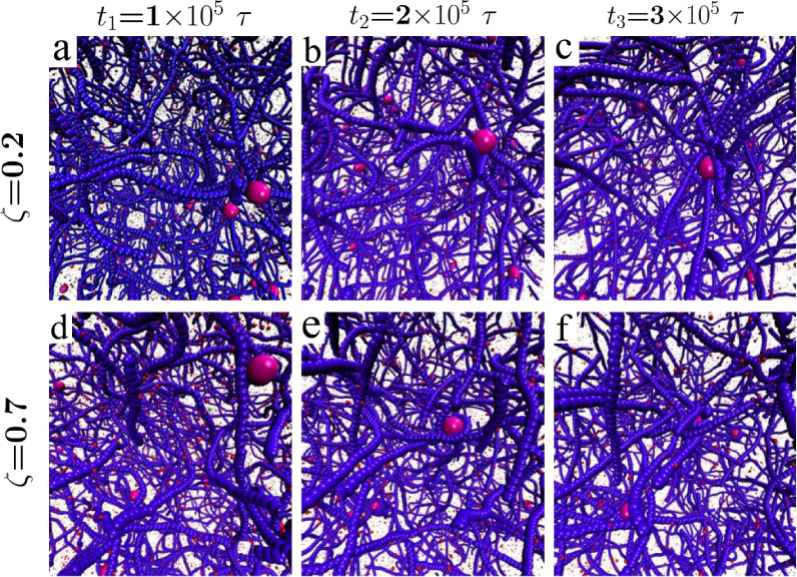
Simulation snapshots depicting the impact of relative charge density on nanoparticle behavior for case of *c* = 2 g/L for case of *D* < ξ. Panels (a–c) display the progression over time for a relative charge density of ζ = 0.2, while panels (d–f) illustrate the dynamics at a higher relative charge density of ζ = 0.7. These sequences reveal that nanoparticles exhibit increased adherence (sticking) to polymers at the higher charge density, indicating a strong dependency of nanoparticle–polymer interaction dynamics on the relative charge density.

In Figure 5, panels a–f present snapshots from simulations illustrating that the diffusion and permeation of NPs within the polymer network play a crucial role in understanding the charge-dependent permeation behavior when the diameter of the NPs is less than the mesh size of the system, *D* < ξ. The same cases for *D* ≈ ξ are shown in SI.

The top row of the figure illustrates the case of ζ = 0.2, showing a relatively low interaction strength between the NPs and the polymer network. Over time, as observed in Figure 5a–c and more vividly demonstrated in Movie S2, the electrostatic attraction drives NPs toward the negatively charged polymers. Initially, as the NPs approach and adhere to the polymers, they settle onto the fibers. However, due to the inherent dynamics of the polymer network, kinetic energy imparts motion to the polymers, which moderately counteracts the electrostatic attraction, causing the NPs to slide along the polymer strands. As the simulation progresses, other negatively charged polymer strands are drawn toward the NPs due to electrostatic forces, leading to their congregation around the NPs.

Consequently, this aggregation results in a marked decrease in the mobility of the NPs. The second row of panels, Figure 5d–f, shows how the system behaves with a higher value of ζ, indicating stronger electrostatic interactions. These snapshots, along with Movie S3, demonstrate the rapid and secure adhesion of NPs to the polymer network. This implies a stronger and more enduring bond than situations with lower ζ values illustrated in the first row. The increased charge density accelerates the attraction between nanoparticles and polymers.

Consequently, reduced sliding of NPs along the polymer strands is observed, leading to a decrease in permeation length. Additionally, the stronger electrostatic interactions prompt adjacent polymers to adhere more securely to the NPs, wrapping them. This is more evident in Figure 5f, where the polymer network nearly fully envelops the nanoparticles. In simulations where *D* < ξ, the mechanisms resemble the biological role of exomers—small vesicles crucial for cellular communication and macromolecule transport. Upon release, exomers bind to and integrate with the extracellular matrix (ECM), facilitating cross-linking and increasing cell rigidity.^44,45^ In our study, a higher ζ value represents a scenario where NPs, like exomers, strongly bind to the fibrous network,^46,47^ resulting in rapid and stable adherence.

**Figure 6:**
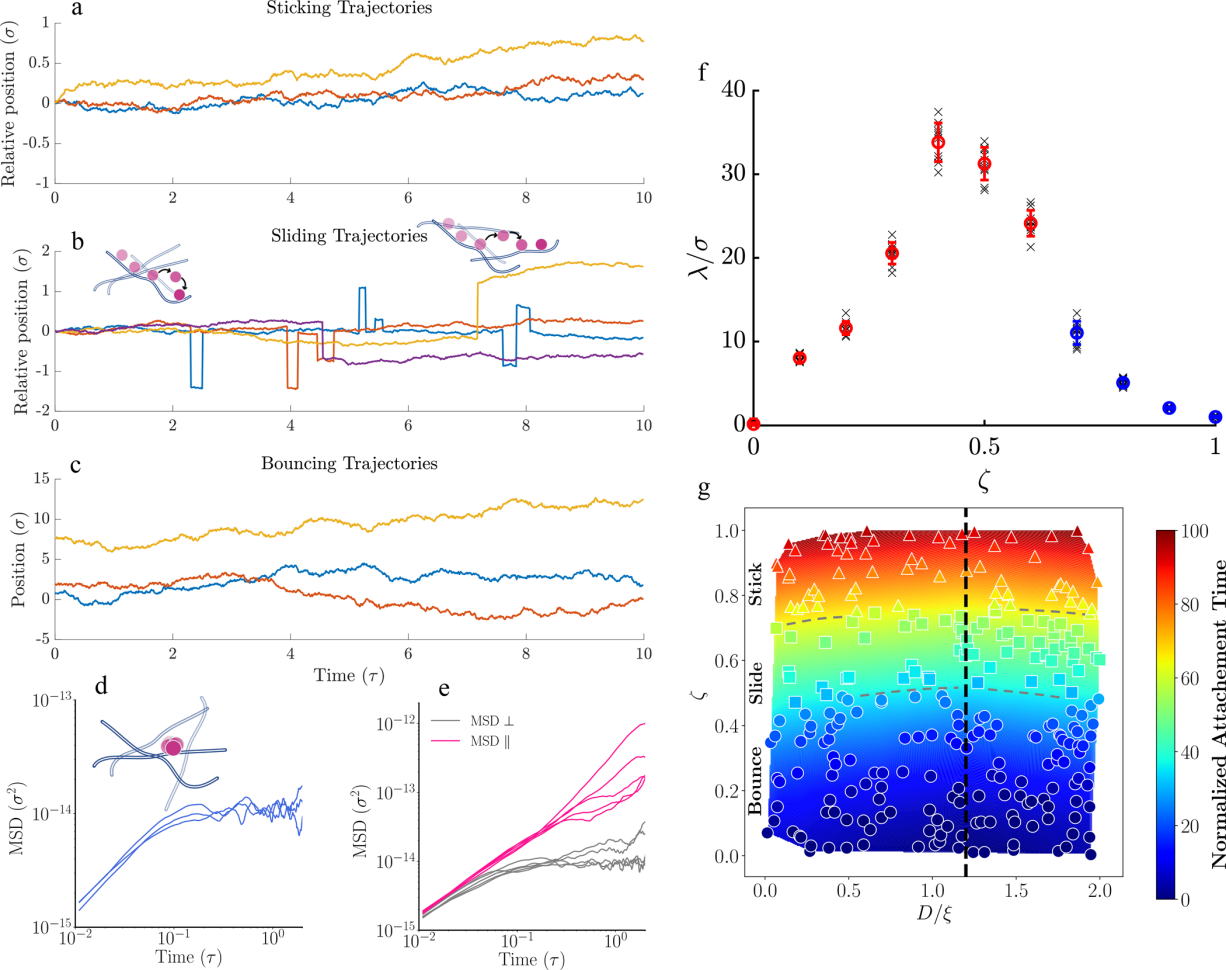
Dynamic analysis of NPs and their binding to polymer chains. (a–c) Trajectory analysis for cases of sticking, sliding, and bouncing, respectively. In sticking regime (a), the particle binds to the polymer chain, as shown by the relative total position of the NP relative to the polymer chain. In sliding regime (b), indicated by the relative normal position of the NP to the polymer chains, NPs slide along the polymer chain and then either jump to a new chain or return to the original chain. In bouncing cases (c), the plotted position does not show a specific pattern. (d) Mean squared displacement (MSD) for sticky NPs. (e) Normal and parallel MSD for sliding particles, showing that while the normal component indicates trapping behavior, the parallel component exhibits linear behavior due to fast sliding. (f) Normalized sliding lengths of NPs to the polymer width versus the ζ parameter. Initially, the sliding length increases with rising ζ values, then drops as sliding turns to sticking behavior. (g) Discrete data points represent the normalized attachment time (NAT) of nanoparticles: circles for NAT < 30 indicate bouncing, squares for 30 ≤ NAT ≤ 60 denote sliding, and triangles for NAT > 60 signify sticking. A color gradient interpolation overlays the data, with varying color intensity reflecting the attachment duration and visualizing the NAT continuum. Boundary lines define the behavioral regimes: bouncing, sliding, and sticking. A vertical dashed line marks the *D*/ξ ratio of 1.2, indicating a regime change in the NAT.

To analyze in detail, the trajectories of the three regimes—sticking, sliding, and bouncing—have been plotted. In Figure 6a–c, the relative positions (a and b) and positions (c) of NPs are shown to illustrate these dynamics. Figure 6a demonstrates the sticking behavior. As seen, the relative position of NPs to the polymer chains shows small fluctuations around zero, indicating that the NPs are bonded or stuck to the chains. In Figure 6d, the mean squared displacement (MSD) of the NPs is plotted, showing that the MSD eventually saturates, indicating that the NPs are indeed bound to the polymer chains. For bouncing cases, the trajectory analysis shown in Figure 6c, indicates a typical Brownian pattern. Figure 6b shows the relative normal positions of NPs with respect to the polymer chains for sliding cases. It is evident that the NPs bind to the polymer, slide for a distance, and then jump or hop. According to the trajectories (Figure 6b), NPs can either jump back to the original polymer chain, shown by blue and orange trajectories, or move to a neighboring one, indicated by purple and yellow trajectories, depending on the network configuration. In Figure 6e, the MSD of sliding particles is divided into two components: normal and parallel to the chain. As shown, the normal component eventually saturates, with a slight increase in some cases due to NPs hopping. In contrast, the parallel component of the MSD, which represents motion along the chain, exhibits increasing behavior.^48^ This mechanism is called facilitated diffusion,^49−53^ as the sliding motion of NPs on the chains follows a fast 1D motion, with a diffusion coefficient potentially up to 18 times higher than the 3D diffusion coefficient of NPs before binding to the chain.^54^ In the simulations, the highest  ratio observed has been approximately 14. In facilitated diffusion, the unbinding rate *k*_off_ is a crucial parameter, defined as *k*_off_ = *P*_off_ τ^–1^, where *P*_off_ is the probability of the NP unbinding from the polymer chain, and τ is the characteristic time of binding.^50^ The sliding length λ can be related to the 1D diffusion coefficient as .

The ratio of sliding length to the polymer width has been calculated and plotted in Figure 4f. Notably, for high ζ cases (shown in blue), where *k*_off_ becomes small number indicating less frequent unbinding and more sticking behavior instead of sliding, the sliding length was calculated manually rather than using the standard relation of As shown, the sliding length for ζ = 0 is almost zero, then increases with ζ, and decreases again to near zero at high ζ values. This indicates that for ζ values approximately between 0.4 and 0.6, the sliding dynamic is the dominant mechanism.

To delve deeper into the sliding and sticking behavior, the average attachment time of NPs to polymer chains is calculated and normalized. Figure 6 presents a complex relationship between normalized attachment time (NAT), relative surface charge density (ζ), and the ratio of the NP diameter to network mesh size (*D*/ξ). First, it is important to note that NAT values less than 30 indicate bouncing, NAT between 30 and 60 denotes sliding, and NAT greater than 60 signifies sticking. The initial observation is the effect of ζ: high ζ values correspond to the sticking region, intermediate ζ values indicate sliding behavior, and lower ζ values are associated with bouncing behavior.

Another noticeable trend is that for ζ values higher than 0.3, where electrostatic interactions become more significant, the behavior changes more distinctly. For example, at ζ = 0.77, as we move from left (small *D*/ξ) to right up to *D*/ξ around 1.2, the NAT value decreases, indicated by the color shift from red/orange to blue/green. Beyond *D*/ξ of approximately 1.2, the NAT value increases again, as shown by the color shift back to red/orange. This trend shows that there is a tug of war between relative NP size (*D*/ξ) and ζ, which affects the NAT. In simpler terms, electrostatic attraction is the dominant parameter in the adherence of smaller NPs to polymer chains. For smaller NPs, high ζ values lead to stronger electrostatic attraction, resulting in a higher NAT and binding behavior. As the size of the NP increases (up to *D*/ξ ≈ 1.2), hydrodynamic and inertial forces begin to offset the electrostatic adherence. In this size range, NPs are more likely to jump or hop from the polymer chains, resulting in lower NAT values. This indicates that intermediate-sized NPs experience a balance between electrostatic attraction and forces that promote movement.

However, when the NP size increases further (*D*/ξ > 1.2), steric hindrance becomes more significant. The larger size restricts the NP’s mobility, causing it to interact more closely with the polymer network. As a result, electrostatic attraction again becomes the dominant factor, leading to higher NAT values and a return to sticking behavior.

This dynamic interplay highlights how both particle size and charge jointly influence the NP’s behavior within the polymer network. Smaller NPs bind due to electrostatic forces, intermediate-sized NPs experience a balance of forces, and larger NPs bind again due to steric hindrance combined with electrostatic attraction. Notably, this pattern is markedly more pronounced at lower zeta values, indicating that at higher charge densities, the sticking behavior is predominantly governed by electrostatic interactions, with NP size playing a secondary role. This detailed examination highlights the delicate interplay between charge density and the physical dimensions of NPs, pivotal in dictating their diffusion within charged polymer networks.

## Conclusions

This study proposes a framework for understanding NP dynamics within polymer networks by combining both numerical simulations and experimental observations. By examining the effects of key parameters, such as NP size, relative surface charge ratio, and environmental concentration, we developed correlations for relative diffusivity and permeation length, specifically accounting for electrostatic interactions. Our analysis of MFPT highlights the critical role of relative surface charge, demonstrating that it is as influential, if not more so, than environmental concentration in determining NP’s fate. Furthermore, we identified distinct dynamic regimes—sticking, sliding, or bouncing—based on the relationship between NP size, mesh size, and charge. These findings advance our understanding of NP dynamics in complex environments and provide a valuable tool for optimizing NP-based drug delivery systems, particularly in designing targeted therapies where timing and precision are crucial.^55−58^

Looking ahead, integrating the effect of pH in simulation algorithms holds promise for further enhancing our understanding of NP behavior under varying pH conditions, which is critical for replicating complex biological environments encountered in vivo.^43^ Thus, this study bridges theoretical simulation and practical applications, driving the field toward innovative solutions in nanoparticle-mediated drug delivery.

## Methods

### Simulation Overview

We conducted comprehensive molecular dynamics (MD) simulations to explore the interactions within a system comprising negatively charged polymers and positively charged nanoparticles (NPs). These simulations were performed using the Extensible Simulation Package for Research on Soft Matter (ESPResSo)^59^ within the canonical (NVT) ensemble.

### Polymer Modeling

In our study, the polymers were represented using the bead–spring model, where the bead sizes were chosen to be 5 nm. Similar to our previous study,^27^ we utilized an attractive potential to precisely represent each polymer chain, employing interconnected MD beads. Specifically, we utilized a standard harmonic interaction to achieve this, formulated as follows:
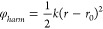
where *r* is the distance between two adjacent MD beads in a polymer chain, *r*_0_ is the equilibrium distance of the potential, and *k* is the spring constant that defines the bond stiffness. The angle potential is used to directly tune the nanofiber bending rigidity as follow
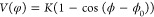
where *K* is bending constant and ϕ_0_ is the equilibrium bond angle in radians ranging from 0 to π.

In assigning charges to the polymers, we drew insights from real-life scenarios involving charged biopolymer systems, such as TEMPO-oxidized cellulose nanofibers, which were also employed in our experiments. We derived this insight from parameters, such as their surface charge density or their zeta potential. Through calculations, we distributed equal amounts of charges among the chains. However, to mirror the heterogeneous nature of biological polymers, we distributed the net charge of the chain unevenly among the beads, allowing for the possibility that a bead may receive zero charge.

### Nanoparticle (NP) Modeling

Positively charged nanoparticles were incorporated into the simulation to investigate their interactions with the charged polymer matrix. The size of these NPs was selected to study their behavior across different regimes: smaller than (*D* < ξ), comparable to (*D* ≈ ξ), and larger than (*D* > ξ) the polymer mesh size (ξ), which is calculated as ξ = √(3/νL), where *v* represents the number of fibers per unit volume. The charge fraction of NPs was adjusted to achieve a surface charge density ratio (ζ) spanning from zero (indicating neutral NPs) to one (signifying that NPs and polymers possess identical surface charge densities).

### Simulation Parameters and Conditions

The simulations are conducted within a periodic box. The selection of the simulation box must be approached with careful consideration as periodic boundary conditions introduce finite-size effects. This is particularly crucial due to the long-range nature of both electrostatic and hydrodynamic interactions within the system, where image particles can interact with one another.

Taking into account the fundamental length scale of the simulations, denoted by σ = 5 nm, the sizes of the nanoparticles (NPs) fall within the range of 5σ < *D* < 40σ, with the maximum fiber length reaching 60σ. Considering the largest hydrodynamic radius in the system alongside the Debye length, the box length is determined as *L* = 68σ. It has been demonstrated that due to the presence of salt and charge neutrality beyond the Debye layer, these interactions are screened over longer length scales.

Studies^60,61^ have revealed that in electrophoresis, the far-field fluid velocity decays as , contrasting with the  decay observed in systems devoid of charges. Consequently, as long as the sum of the Debye length and the hydrodynamic radius remains smaller than , the finite size effects can be considered negligible.

The fundamental units for energy and mass in MD simulations are represented by ε = *k*_B_*T* and *m*_0_, respectively. Here, *m*_0_ denotes the mass of an ion, approximately 10^–26^ kg, and all ions and monomers are assigned a mass of *m* = 1 *m*_0_ The fundamental unit of time in MD simulations, denoted by τ, is derived from these length, mass, and energy units as .

Interaction forces between particles, including Weeks–Chandler–Andersen (WCA) potentials for excluded volume effects,
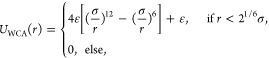


In the system, alongside the charged polymers and NPs, we introduce counterions as hard spheres to maintain the system’s overall charge neutrality. The Debye length of the system can be calculated using
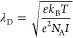


Where ε is the permittivity of the medium, *k*_B_ is the Boltzmann constant, *T* is the temperature in Kelvin, *e* is the elementary charge, *N*_A_ is Avogadro’s number, and *I* is the ionic strength of the solution.

In a neutralized system with no added salt, the ions present are primarily those associated with the charged polymers, spherical particles, and their counterions. The ionic strength (*I*) in such a scenario can be calculated using
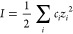


Where *c*_*i*_ is the concentration of ions in mol/L and *z*_*i*_ is the charge number of ions. This sum only accounts for the ions necessary to balance the charges of the polymers and particles, leading to a relatively low ionic strength compared to a system where additional ions have been introduced through salt. Adding salt to the system introduces additional ions, significantly increasing the ionic strength. Higher ionic strength results in a shorter Debye length, indicating more effective screening of electrostatic interactions among charged entities. This can have profound effects on the physical properties of the system, such as reducing repulsion between like-charged entities and potentially promoting stability or aggregation depending on the balance of forces at play.

P3M algorithms^62^ for electrostatic interactions in Espresso have been used. The accuracy level for electrostatic force calculations via the P3M algorithm was maintained at the same level of  as described,^63^ ensuring precise and reliable simulation results.

Hydrodynamic forces were considered by employing the Lattice-Boltzmann Method (LBM) within ESPResSo. We utilized the D3Q19 lattice–Boltzmann (LB) method^64^ as implemented in ESPResSo, with an LB-lattice constant of *a* = 1σ and a time step of *τ*_*LB*_ = 0.001τ. The LB fluid was characterized by a density of  and a kinematic viscosity of . All particles were dissipatively coupled to this background lattice-fluid via a bare coupling constant of 15σ*m/*τ.

## Experimental Methods

### Preparation of TEMPO-Oxidized Cellulose Nanofibers (CNFs)

The TEMPO-oxidized cellulose nanofiber (CNF) suspensions were meticulously prepared, based on Weishaupt et al.,^65^ to establish a fibrous network composed of these biopolymers. We selected a concentration range from 0.2 to 1 g/L for our experiments, a decision driven by our objective to thoroughly investigate how varying concentrations of CNFs influence the interaction and distribution of dyes within the fibrous structure. This careful selection is pivotal for understanding the dynamics of dye absorption and its uniformity across the nanofiber network.

Initially, the CNFs were dispersed in Milli-Q water, thereby providing a consistent basis for evaluating dye interactions. The suspension was then subjected to a rigorous homogenization process. This step was crucial for achieving a homogeneous dispersion of the nanofibers, thereby guaranteeing consistent results across all experimental runs.

To corroborate the uniformity and structural integrity of the prepared TEMPO-oxidized CNFs, we conducted comprehensive characterizations. These included atomic force microscopy (AFM) analysis, scanning electron microscopy (SEM) imaging, and DLS and zeta potential analysis. The findings from these characterizations, detailed in Figures S1 and S2 and Table S1, provide insight into the nanofibers’ morphology and the efficacy of the preparation process. Through these analytical techniques, we were able to confirm the successful preparation of the CNF suspensions and their readiness for further experimentation in studying dye interactions within the fibrous network.

### Preparation of Dyes for Interaction Studies with CNF Suspension

We selected three dyes, each of which was characterized by distinct charge properties. This selection included Congo red, which carries a negative charge, and both thionine and methylene blue, which are positively charged. The choice of these dyes was driven by the objective to comprehensively understand how the charge characteristics of different dyes influence their interactions and distributions within the CNF suspension.

To ensure uniformity and comparability across all experimental conditions, each dye was dissolved in an aqueous medium to a precise concentration of 3 mM.

Recognizing the crucial role of pH in charge interactions, we meticulously adjusted the solutions of both the CNF suspensions and the dyes to a neutral pH. This step was crucial for eliminating pH-induced variations in charge behavior, thus ensuring that any observed interactions could be solely attributed to the inherent properties of the CNFs and the dyes.

Dynamic light scattering (DLS) analysis (Table S1) was subsequently performed to characterize the dyes under these neutral conditions, providing a clear baseline for understanding how their molecular size and distribution influence their behavior in the CNF matrix.

### Atomic Force Microscopy (AFM)

Atomic force microscopy (AFM) measurements were performed on a Bruker Icon3 AFM. First, a droplet of 3-aminopropyl triethoxysilane (APTES) was put on a mica sheet and incubated for 1 min. After functionalization the mica was rinsed with Milli-Q water and air-dried. Then the cellulose nanofibers (CNF) suspension at 1.4 wt % was diluted with Milli-Q water to reach a wt % of 0.001. Afterward, a droplet of the diluted CNF suspension (0.001 wt %) was put on the (APTES) functionalized mica and incubated for 1 min. Again, the sample was rinsed with Milli-Q water and air-dried. The measurements were conducted with RTESPA-150 tips for tapping in air in soft tapping mode with a vibration frequency of 150 kHz. AFM images were post-processed with Gwyddion. The background was flattened, and the minimal value of the scale was set to zero.

### Scanning Electron Microscopy (SEM)

For scanning electron microscopy (SEM) the CNF suspension 1.4 wt % was first diluted with Milli-Q to 0.14 and 0.014 wt %. Then a drop of the diluted CNF suspensions were placed on a mica sheet which was place on a pin stup (alumminium). The pins are then placed in a sputter coater (Baltec MED020) and dried under vacuum for 15 min. Afterward, the sample is coated with platinum (thickness of the coating 7 nm).

### Dynamic Light Scattering (DLS)

DLS and zeta potential measurements were performed with a Zetasizer FX-C13 from Malvern Panalytical. For size measurements disposable 70 μL UV-cuvettes micro were used, and for zeta potential measurements DTS1070 cell from Malvern were used. First, the CNF suspension at 1.4 wt % was diluted to 0.01 wt %. This dilution was then used to measure the size and zeta potential of the CNF suspension. The three different dyes Congo red, methylene blue, and thionine were diluted to concentrations of 0.007, 0.005, and 0.005 mM, respectively, and their zeta potential was measured.

For the experiments, the CNF suspension was transferred to quartz cuvettes. These cuvettes were chosen for their transparency and chemical inertness, qualities essential for subsequent analytical procedures.

## Supporting Information Available

Setup for analyzing dye–CNF interactions; image capture and analysis of dye dispersion; table showing the zeta potential of samples; atomic force microscopy (AFM) images of CNF samples; scanning electron microscopy (SEM) images of CNF samples; raw images of dye spreading in the CNF system alongside their postprocessed images; the illustration of separate effect of relative charge and concentration on relative diffusion; simulation snapshots depicting the impact of relative charge density on nanoparticle behavior for the case of *c* = 2 g/L and *D* ≈ ξ; and comparision of MSDM, free volume, and obstruction theories with fitted lines to simulation data for mesh sizes of 30, 20, and 10 nm (PDF)
The diffusion of an NP within a dynamic mucus structure (*D* < ξ) (MP4)
The process of thionine release in a network over a 2 min period (MP4)
The process of Congo red release in a network over a 2 min period (MP4)
The rupture of circular membrane at high charge density difference (MP4)
A detailed observation of how the concentration of the polymer network and the positive charge of NPs influence the dispersion and symmetry of NPs within the system(MP4)
The process how NPs are attracted to the dynamic polymers at *D* < ξ (MP4)
The process how NPs are attracted to the dynamic polymers at *D* = ξ (MP4)
The process how NPs are attracted to the dynamic polymers at ζ = 0.2 (MP4)
The process how NPs are attracted to the dynamic polymers at ζ = 0.7 (MP4)
