# Supplementary material for: Stick, Slide, or Bounce: Charge Density Controls Nanoparticle Diffusion
Source: ACS Nano. 2024 Oct 8;18(42):28636–48. doi: 10.1021/acsnano.4c05077 (PMC11503907; doi:10.1021/acsnano.4c05077)
Supplement: Supplementary file 1 — nn4c05077_si_001.pdf [file nn4c05077_si_001.pdf]

Stick, slide or bounce: charge density controls nanoparticle diffusion

Ahmad Reza Motezakker[†,‡], Luiz G. Greca[¥], Enrico Boschi[¥], Gilberto Siqueira[¥], Fredrik Lundell[†], Tomas Rosén[‡,¶], Gustav Nyström[¥,¤], L. Daniel Söderberg[‡,¶]

† Department of Engineering Mechanics, KTH Royal Institute of Technology; Stockholm, SE-100 44, Sweden

‡ Wallenberg Wood Science Center, KTH Royal Institute of Technology; Stockholm, SE-100 44, Sweden

¥ Laboratory for Cellulose and Wood Materials, Swiss Federal Laboratories for Materials Science and Technology (Empa), Dübendorf, 8600 Switzerland

¤ Department of Health Sciences and Technology, ETH Zürich, Zürich, 8092 Switzerland

¶ Department of Fibre and Polymer Technology, KTH Royal Institute of Technology; Stockholm, SE-100 44, Sweden

Setup for Analyzing Dye-CNF Interactions

To study how different dyes interact with TEMPO-oxidized cellulose nanofiber (CNF) suspensions, we used a contact angle measurement device, model TBU 90E from Dataphysics. We carefully added 1 microliter of each dye to the CNF suspension in quartz cuvettes. For this, we used a syringe with a 0.5 mm needle, ensuring that we introduced the dye gently and accurately. This method helped us avoid disturbing the CNF network too much and allowed for precise measurements of how the dye spread within the suspension. This approach was crucial for our analysis, as it let us closely observe the behavior of the dyes when they came into contact with the CNFs, giving us clear insights into the interaction patterns between them.

Image Capture and Analysis of Dye Dispersion

The process of dye dispersion within the TEMPO-oxidized cellulose nanofiber (CNF) suspensions was monitored in real time. This was achieved using a high-resolution camera integrated with the contact angle measurement device. The purpose was to visually capture the dye's spread and its interactions with the CNF network as they occurred. For the analysis of these images, we employed ImageJ for initial processing, followed by further analysis with Python. This two-step approach allowed us to quantify the dynamics of the dye dispersion, its interaction with the CNFs, and the stability of these interactions over time. A notable aspect of our methodology was the use of the 0.51 mm needle, which served as a scale bar in our images. This reference enabled us to upscale the image quality accurately, apply pixelation techniques, and, by correlating pixel size with the scale bar, estimate the size of features within the images. Through image processing techniques, we were able to detect edges and outline the contours of the spreading dye. This also included measuring the growth rate and size of the dye patches. Such detailed analysis provided us with significant insights into the physicochemical interactions between the dyes and the CNF network. Understanding these interactions is crucial for unraveling the material's properties and exploring its potential applications. A sequence of processed images is shown in Figure S3.

Table. S1. Zeta potential of samples

Samples	Zeta potential (mV)
T-CNF	-59.3
Congo Red	-18.7
Methylene Blue	1.85
Thionine	30.6

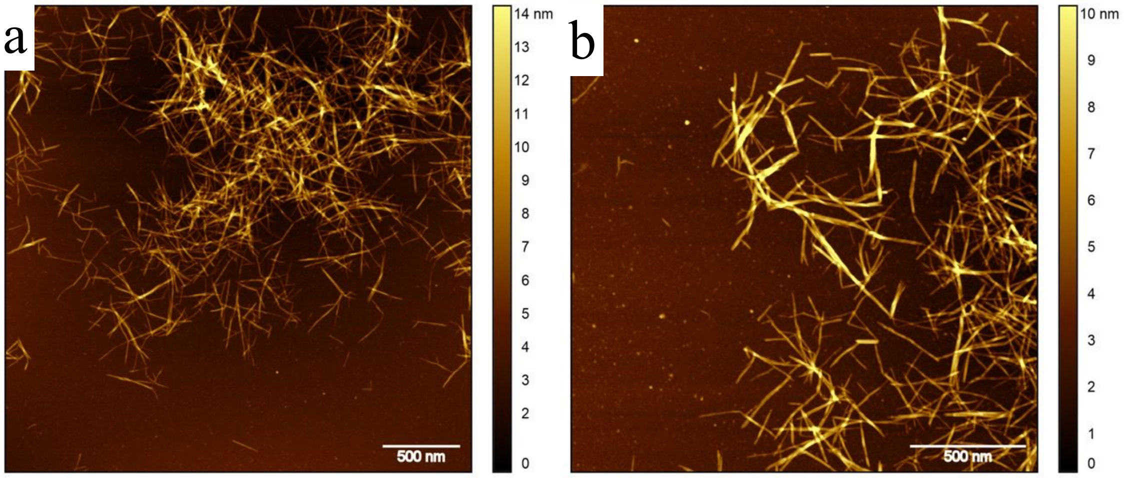

Figure S1. Atomic force microscopy (AFM) of CNF samples

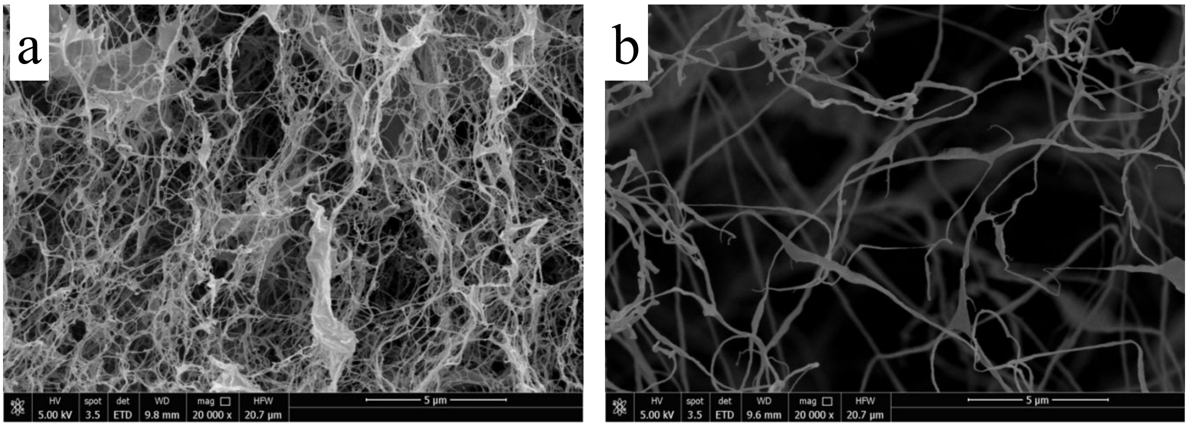

Figure S2. Scanning electron microscopy (SEM) of CNF smaples

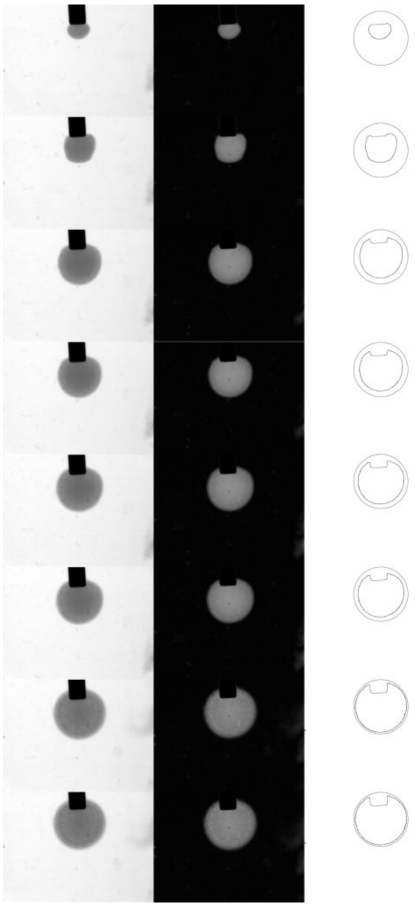

Figure S3. Raw images of dye spreading in the CNF system beside their post processed images

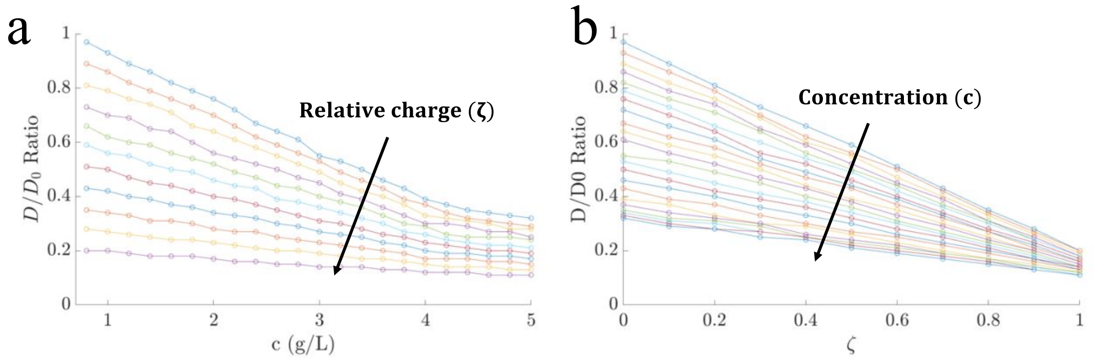

Figure S4. Separate effect of relative charge and concentration on relative diffusion

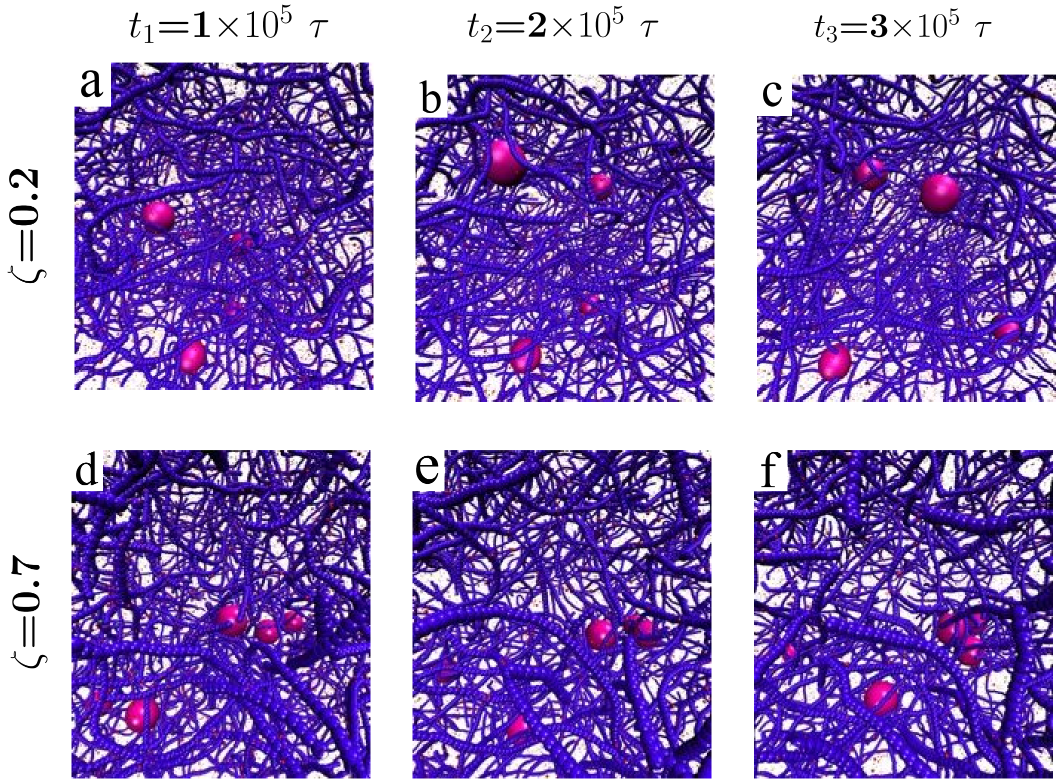

Figure S5. Simulation snapshots depicting the impact of relative charge density on nanoparticle behavior for case of c = 2 g/L for case of D~ ξ.

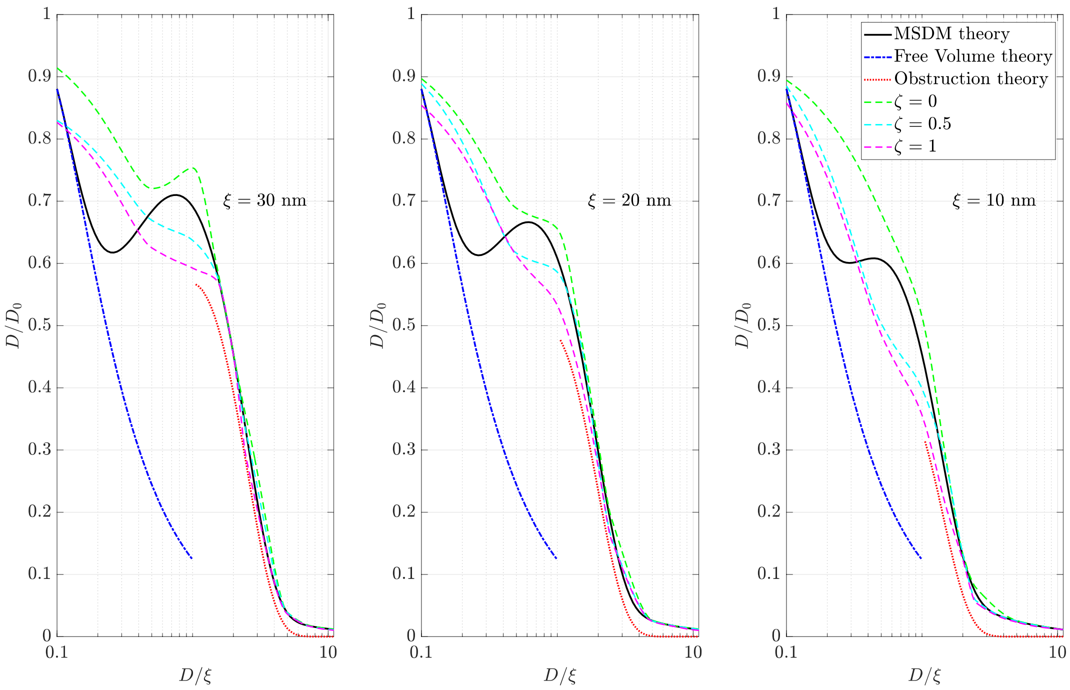

Figure S6. Comparison of MSDM, Free Volume, and Obstruction theories with fitted lines to simulation data for mesh sizes of 30, 20, and 10 nm.
